# Are animal models predictive for humans?

**DOI:** 10.1186/1747-5341-4-2

**Published:** 2009-01-15

**Authors:** Niall Shanks, Ray Greek, Jean Greek

**Affiliations:** 1 Wichita State University, Department of History, 1845 N Fairmont, Fiske Hall, Wichita KS 67260, USA; 2 Americans For Medical Advancement, 2251 Refugio Rd Goleta, CA 93117, USA

## Abstract

It is one of the central aims of the philosophy of science to elucidate the meanings of scientific terms and also to think critically about their application. The focus of this essay is the scientific term *predict *and whether there is credible evidence that animal models, especially in toxicology and pathophysiology, can be used to predict human outcomes. Whether animals can be used to predict human response to drugs and other chemicals is apparently a contentious issue. However, when one empirically analyzes animal models using scientific tools they fall far short of being able to predict human responses. This is not surprising considering what we have learned from fields such evolutionary and developmental biology, gene regulation and expression, epigenetics, complexity theory, and comparative genomics.

## Review

"When I use a word,' Humpty Dumpty said in rather a scornful tone, 'it means just what I choose it to mean – neither more nor less." "The question is," said Alice, "whether you can make words mean so many different things."

Lewis Carroll in *Through the Looking Glass *1871.

There is a serious scientific controversy concerning the predictive power of animal models. In this article we will use the phrase *animal model *to mean, or the word *animal *in the context of, the use of a nonhuman animal, usually a mammal or vertebrate to predict human response to drugs and disease. We enthusiastically acknowledge that animals can be successfully used in many areas of science, such as in basic research, as a source for replacement parts for humans, as bioreactors and so forth. These uses are not included in our definition or critique as there is no claim made for their predictive power. This article focuses solely on using animals/animal models to predict human responses in light of what the word *predict *means in science.

### Philosophy of science

The meaning of words is very important in all areas of study but especially science.

Philosophers of science including Quine, Hempel and others have argued that words must have meaning in science and in fact these meanings separate science from pseudoscience. Take for example the word *prediction*. A research method need not be predictive to be used but if one claims predictive ability for the test or project, then one means something very specific.

This paper addresses the use of the word *predict *as applied to animal models. It is our position that the meaning of the word has been corrupted and hence the concept behind the word is in danger as well as everything the concept implies. *Predict *is not so much a word as a concept and is closely related to *hypothesis*. Hypothesis can be defined as a proposed explanation for a phenomenon, either observed or thought, that needs to be tested for validity. According to Sarewitz and Pielke:

In modern society, prediction serves two important goals. First, prediction is a test of scientific understanding, and as such has come to occupy a position of authority and legitimacy. *Scientific hypotheses are tested by comparing what is expected to occur with what actually occurs*. When expectations coincide with events, it lends support to the power of scientific understanding to explain how things work. " [Being] predictive of unknown facts is essential to the process of empirical testing of hypotheses, the most distinctive feature of the scientific enterprise," observes biologist Francisco Ayala (Ayala, F. 1996. The candle and the darkness *Science *273:442.)[1] (Emphasis added.)

In the case of animal models *what actually occurs *is what happens in humans. If the purpose of the test, be it a test on an animal or *in silico*, is to predict human response then the tests must be evaluated by how well it conforms to human response. We again acknowledge that not all tests and studies involving animals are done with prediction in mind. Nevertheless, those tests promoted as being predictive must be judged by how well they predict human response.

Sarewitz and Pielke continue:

Second, prediction is also a potential guide for decision making. We may seek to know the future in the belief that such knowledge will stimulate and enable beneficial action in the present [1].

We will return to *decision making*.

The philosopher W.V.O. Quine has remarked:

A prediction may turn out true or false, but either way it is diction: it has to be spoken or, to stretch a point, written. Etymology and the dictionary agree on this point. The nearest synonyms "foresight," "foreknowledge," and "precognition" are free of that limitation, but subject to others. Foreknowledge has to be true, indeed infallible. Foresight is limited to the visual when taken etymologically, and is vague otherwise. "Precognition" connotes clairvoyance ... *Prediction is rooted in a general tendency among higher vertebrates to expect that similar experiences will have sequels similar to each other *[[2]159] ... (Emphasis added.)

Predictions, generated from hypotheses, are not always correct. But if a modality or test or method is said to be predictive then it should get the right answer a very high percentage of the time in the biological sciences and all the time in the physical sciences. (We will discuss this as it applies to the biological sciences momentarily.)

If a modality consistently fails to make accurate predictions then the modality cannot be said to be predictive simply because it occasionally forecasts a correct answer. The above separates the scientific use of the word *predict *from the layperson's use of the word, which more closely resembles the words *forecast, guess, conjecture, project *and so forth. We will return to these points shortly

Many philosophers of science think a theory (and we add, a modality) could be confirmed or denied by testing the predictions it made. Unlike the physical sciences, the biological sciences, which study complex systems, must rely on statistics and probability when assessing what the response to a stimulus will be or in discussing the likelihood a phenomenon will occur. There is an assumption that has taken on the trappings of a theory or perhaps an overarching hypothesis in the animal model community that results from experiments on animals can be directly applied to humans; that animal models are predictive. This has resulted in an unquestioned methodological approach; using animals as surrogate humans. Ironically, this hypothesis has not been questioned as hypotheses should be questioned in science, hence our calling it an *overarching *hypothesis. Whether or not the animal model *per se *can be used to predict human response *can *be tested and if the results have a high enough sensitivity, specificity, positive and negative predictive value then the hypothesis that animals can predict human response would be verified. If verified, then one could say that animal models are predictive for humans and if refuted then one could say animal models are not predictive for humans.

There are two very different ways animals and hypotheses are used in science. Hypotheses result in predictions that have to be tested thereby confirming or falsifying the hypothesis. Let us assume that a scientist is using animals to perform basic research. At the end of the series of animal experiments the investigator has, at most, a hypothesis about a likely human response to the same stimulus or substance, when allowances have been made for differences in body weight, exposure, and so on. The prediction that the hypothesis entails must then be tested, and this will require the gathering of human data. The prediction may be verified or it may be falsified in the light of such human data, but the evidential burden here cannot be evaded from the standpoint of basic scientific methodology. Nowhere in this use of animals to generate a hypothesis have animals been assumed predictive. LaFollette and Shanks have referred to the practice of using animals in this fashion as heuristic or hypothetical animal models (HAMs) [3,4].

This is in contrast to the hypothesis that some scientists start with, namely that animals are predictive for humans. (See table 1.) By assuming this, these scientists make the claim that drugs and chemicals that would have harmed humans have been kept off the market secondary to results from animal tests. This is disingenuous unless we have *a priori *reason to assume animal models are predictive. The hypothesis was, in these cases, never tested. It would in many cases be unethical to conduct on humans the sorts of carefully controlled laboratory studies that are regularly conducted on, say, rodents. However, there are other, ethical ways to gain human data in the context of epidemiology (for example retrospective and prospective epidemiological studies), *in vitro *research using human tissue, *in silico *research, and the recent technological breakthrough of microdosing [5]. Further, it must never be forgotten that when industrial chemicals find their way into the environment, or drugs are marketed to the general population, human experiments have already taken place. Moreover, as Altman [6] has observed, there are many examples, both ancient and modern, where researchers, doubting the applicability or relevance of animal models to the human situation, have experimented on themselves – a practice that Altman points out continues to the present (recent Nobel laureate Barry Marshal being but one example). In any event, at the very least a track record of success (vis-à-vis positive and negative predictive values) using specific animal models should be evident if society is to accept hypotheses from animal testing as predictive for humans.

**Table 1:** Hypothesis

	Animal models in toxicology and disease research.	Correct use of animal models in areas such as basic research.
Assumptions	Usual plus animal models are predictive.	Usual, e.g. there are universal laws.
Hypothesis	X leads to Y.	None or X leads to Y.
Animal test	In an animal test, X led to Y.	X leads to Y based on results in animals.
Hypothesis		X leads to Y in humans.
Test		Apply X in humans and see if it leads to Y or study populations where X was applied and ascertain result.
Conclusion	Since in animals X did/did not lead to Y, X will/will not lead to Y in humans also.	Actual results from humans.

Therefore, we must emphasize that when discussing animals as predictive models we are discussing the overarching hypothesis that animals are predictive, not the use of animals to generate a hypothesis such as occurs in basic research.

Now is an appropriate time to discuss the concept of proof and where the burden of proof lies in science. As in law, it lies on the claimant. The null hypothesis demands that we assume there is no connection between events until such causation is proven. Thus, those claiming animal models are predictive of human responses in the context of biomedical research must show that what they are claiming is true. The burden is not on us to prove that animal models of, say carcinogenesis or toxicity, are *not *predictive. It is the job of those advocating animal models as predictive to demonstrate they are. This will require a consideration of what the evidence actually shows.

While physics deals with simple systems upon which reductionism can be practiced, biology does not always have that luxury. There are areas in biology – for example comparative anatomy – where the use of scaling principles have had genuine applicability. But biology is not physics, and there are other places – for example in some branches of predictive toxicology – where the use of such scaling factors (such as body weight^2/3^) have been less than useful for reasons we will explore below. Biological systems are certainly consistent with the laws of physics, but they have properties consequent upon internal organization, ultimately rooted in evolutionary history, not found in physics. This means that even when the same stimulus is applied, end results can differ markedly. The response of different humans to the same drug or disease is a well-known example of this phenomenon [7-13]. There are however, ways to judge the predictive nature of tests even in biological complex systems. Values such as positive predictive value, sensitivity, specificity, and negative predictive value (we will discuss these values momentarily) can be calculated to confirm or refute hypotheses. Values from tests seeking to predict a response that approach what would be expected from random chance would obviously not fall into the category *predictive*.

### Claims about the predictive nature of animal models

According to Salmon there are at least three reasons for making predictions:

1. because we want to know what will happen in the future;

2. to test a theory;

3. an action is required and the best way to choose which action is to predict the future [14].

In the case of carcinogenesis we want to know: (1) what will happen in the future (will the chemical cause cancer in humans?); and (3) an action is required (allow the chemical on the market or not?) and the best way to choose which action is to be able to predict the future. Neither (1) nor (3) is subtle. We want a correct answer to the question, "Is this chemical carcinogenic to humans?" or to similar questions such as, "What will this drug do to humans?" and "Is this drug a teratogen?" and "Is this the receptor used by HIV to enter the human cell?" But guessing correctly or finding correlations are not, as we have seen the same as predicting the answer. Neither is a high degree of sensitivity alone, as we shall see, the same as prediction.

The following will help the reader gain a feel for the contours of this scientific debate.

Butcher [15], Horrobin [16], Pound et al. [17] and others [3,4,18-24] have questioned the value of using animals to predict human response. Regardless, prediction is a problem. U.S. Secretary of Health and Human Services Mike Leavitt stated in 2007:

Currently, nine out of ten experimental drugs fail in clinical studies because we cannot accurately predict how they will behave in people based on laboratory and animal studies" [24].

This is a very damaging statement for those who assert that animals are predictive. For some, the facts behind this statement would, without further support, answer the prediction question. But we will continue.

This debate has recently expanded to *Philosophy, Ethics, and Humanities in Medicine*. Knight [25] recently questioned the use of chimpanzees in biomedical research citing among other reasons their lack of predictability. Shanks and Pyles [26] questioned the ability of animals to predict human response resulting in Vineis and Melnick [27] responding that animals can be used to predict human response to chemicals with reference to carcinogenesis and that epidemics of cancer could have been prevented if animal data had been used to reduce human exposure or ban the chemical entirely. This claim, of animals predicting human response, is not unique [28,29].

Gad wrote in *Animal Models in Toxicology *2007:

Biomedical sciences' use of animals as models to help understand and *predict *responses in humans, in toxicology and pharmacology in particular, remains both the major tool for biomedical advances and a source of significant controversy ...

At the same time, although there are elements of poor practice that are real, by and large animals have worked exceptionally well as *predictive *models for humans-when properly used ...

Whether serving as a source of isolated organelles, cells or tissues, a disease model, or as a *prediction *for drug or other xenobiotic action or transformation in man, experiments in animals have provided the necessary building blocks that have permitted the explosive growth of medical and biological knowledge in the later half of the 20th century and into the 21st century ...

Animals have been used as models for centuries to *predict *what chemicals and environmental factors would do to humans ... The use of animals as *predictors *of potential ill effects has grown since that time [the year 1792].

Current testing procedures (or even those at the time in the United States, where the drug [thalidomide] was never approved for human use) *would have identified the hazard and prevented this tragedy *[29]. (Emphasis added.)

Fomchenko and Holland observe:

GEMs [genetically engineered mice] closely recapitulate the human disease and are used to *predict *human response to a therapy, treatment or radiation schedule [30]. (Emphasis added.)

Hau, editor of an influential handbook on animal-based research notes:

A third important group of animal models is employed as *predictive *models. These models are used with the aim of discovering and quantifying the impact of a treatment, whether this is to cure a disease or to assess toxicity of a chemical compound [31].

Clearly, Hau offers the use of animals as predictive models just as we are describing.

The prediction claim is also strong when the word prediction is not actually used but is implied or linked to causality. Fomchenko and Holland continue:

Using *in vitro *systems and in vivo xenograft brain tumor modeling provides a quick and efficient way of testing novel therapeutic agents and targets, knowledge from which can be translated and tested in more sophisticated *GEMs that faithfully recapitulate human brain tumors and will likely result in high-quality clinical trials with satisfactory treatment outcomes and reduced drug toxicities*. Additional use of GEMs to establish *causal links *between the presence of various genetic alterations and brain tumor initiation or determining their necessity for tumor maintenance and/or progression provide us with a glimpse into other important aspects of brain tumor biology [30]. (Emphasis added.)

Fomchenko and Holland are here clearly saying what happens in animals will happen in humans; that animals are predictive. Akkina is saying the same:

A major advantage with this in vivo system [genetically modified SCID mice] is that any data you get from SCID-hu mice is directly applicable to a human situation [32].

This use of prediction is not confined to the scientific literature. It is, if anything even more widespread when scientists are speaking to the nonscientist public.

The above examples could be multiplied without effort. Due to the ubiquitous nature of comments like the above, we can safely deduce that many in the scientific community use the word *predict *to mean that what happens in animal models will translate directly to humans. But is this a factual interpretation of reality?

### Prediction in biological complex systems

What does constitute prediction in biological complex systems? Many justify the use of animals as predictive models by stating that animals are predictive but may not be reliably predictive. This seems to be oxymoronic. *Reliably predictive *would be a tautology and a method cannot be predictive, in science, if it is not reliably so. However, we acknowledge that biology is not physics so perhaps some leniency is needed when discussing prediction in biological complex systems. How then should we think of prediction in the context of toxicology, pathophysiology, and pharmacology? The 2 × 2 table for calculating sensitivity, specificity, positive predictive value and negative predictive value is how predictability is assessed in these contexts (see table 2).

**Table 2:** Statistics used in analysis of prediction.

		Gold Standard	
		+	-
Test	+	TP	FP
	-	FN	TN

TP = True positive

TN = True negative

TN = True negative

FN = False negative

Sensitivity = TP/TP+FN

Specificity = TN/FP+TN

Positive Predictive Value = TP/TP+FP

Negative Predictive Value = TN/FN+TN

In biology many concepts are best evaluated by using simple statistical methods involving probability. For example, in medicine we can use a blood test to determine whether someone has liver disease. In order to ascertain how well this test actually determines the health of the liver we calculate the sensitivity and specificity of the test along with the positive predictive value (PPV) and negative predictive value (NPV). The sensitivity of a test is the probability (measured on a scale from 0.0 to 1.0) of a positive test among people whose test should be positive – those who do in fact suffer from liver disease. Specificity is the probability of a negative test among people whose test should be negative – those without liver disease. The positive predictive value of a test is the proportion of people with positive test results who are actually positive. The negative predictive value is the proportion of people with negative test results who are actually negative. This is all quite straightforward. Very few tests have a sensitivity or specificity of 1.0 or a PPV and NPV of 1.0 but in order for a test to be useful given the demanding standards of medical practice, in this case tell us if the patient actually has liver disease, it needs to be have PPV and NPV in at least the .95 to 1.0 range.

By definition, when we speak of animals predicting human response in drug testing and disease research we are addressing the risks of wrong predictions and how much risk society is willing to tolerate. Troglitazone (Rezulin™) is a good example of the margin of error for medical practice tolerated in society today. Troglitazone was taken by well over 1 million people with less 1% suffering liver failure, yet the drug was withdrawn because of this side effect [33]. (Interestingly, animal studies failed to reproduce liver failure from troglitazone [34].) Rofecoxib (Vioxx™) is another example of the small percentage of morbidity or mortality tolerated in the practice of medicine vis-à-vis introducing a new drug. Figures vary, and are controversial, but it now appears that apparently less than 1% of people who took rofecoxib experienced a heart attack or stroke as a result, yet it was also withdrawn [35]. This means that even if a test with a PPV of .99 had assured industry that rofecoxib and troglitazone were safe, the test would not have been accurate enough for society's standards. This is an important point. Medical practice does not tolerate risks (probability of being wrong) acceptable in some experiments conducted in labs. In basic research we might proceed with a study based on the outcome being more likely than not. For basic research this is acceptable. However, getting the answer wrong in medical practice has consequences; people die. Societal standards for medical practice today demand very high sensitivity, specificity, PPV and NPV from its tests. We will apply the above to animal models shortly.

These standards of prediction, described above, should not be confused with those of other activities in society such as gambling in Las Vegas. If we worked out a method to be correct 51% of the time, we would gladly take that predictive ability to the blackjack table, the roulette wheel, and the craps table and leave with a fortune. Sometimes being correct 51% of the time is great!

In light of the above, it is common to use multiple tests when attempting to determine a patient's condition or evaluate a drug. If someone suggests that an animal, say a mouse, can predict human response to chemicals vis-à-vis carcinogenesis, he would need to provide data consistent with that needed for table 2. Perhaps not one animal alone is capable of predicting human response but when the same result occurs in two species, say and mouse and a monkey, then perhaps the results are predictive. Or perhaps animal data combined with other data translates into a high predictive value. Again, if this were the case the person making the claim should be able to provide data amenable to evaluation by the gold standard laid out in table 2. To the best of our knowledge no such data exists.

### Predicting human response

We will now discuss the actual data that does exist. The data from testing six drugs on animals was compared with the data from humans [36]. The animal tests were shown to have a sensitivity of 0.52 and the positive predictive value was 0.31. The sensitivity is about what one would expect from a coin toss and the PPV less. Not what is considered predictive in the scientific sense of the word. Values of this nature are more appropriately referred to as guesses. Because of data like this, animal modelers will occasionally use the phrase *concordance rate *or *true positive concordance rate *when judging animal tests. These terms are not in the normal prediction-relevant lexicon and are usually used to mean correlation, which has nothing to do with prediction, as we will see.

Two studies from the 1990s revealed that: (1) in only 4 of 24 toxicities were found in animal data first [36]; and (2) in only 6 of 114 cases did clinical toxicities have animal correlates [37]. The sensitivity, specificity, PPV and NPV of animal models based on these studies are obviously suboptimal.

A 1994 study of 64 marketed drugs conducted by the Japanese Pharmaceutical Manufacturers Association found that 39/91 (43%) clinical toxicities were not forecast from animal studies [38]. (This study, as do many others, counted as a positive prediction when *any *animal correlated with the human response. This is disingenuous as it is cherry picking the data.) Without knowing the raw data it is impossible to calculate a true PPV and NPV but even taken at face value, 43% wrong/57% correct is not predictive.

**Figure 1:**
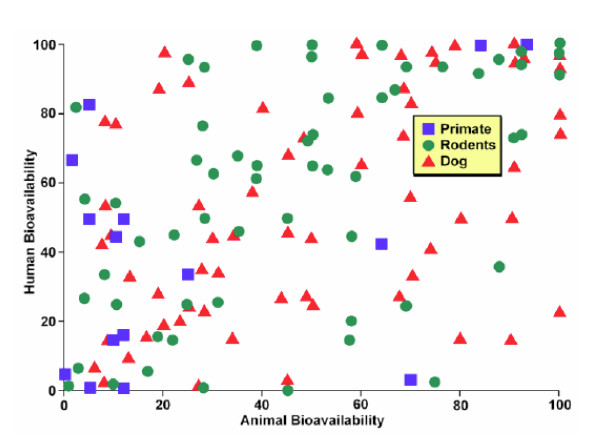
**Human vs animal bioavailability 1**. Graph generously provided by James Harris PhD, who presented it at the Center for Business Intelligence conference titled *6^th ^Forum on Predictive ADME/Tox *held in Washington, DC September 27–29, 2006 and is adapted from data that appeared in Grass GM, Sinko PJ. Physiologically-based pharmacokinetic simulation modelling. Adv Drug Deliv Rev. 2002 Mar 31;54(3):433–5.

**Figure 2:**
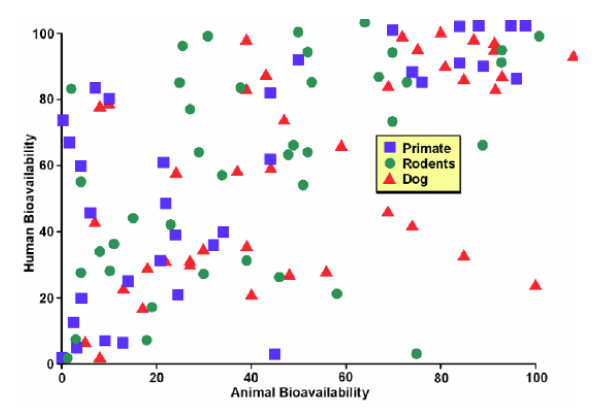
**Human vs animal bioavailability 2**. Graph generously provided by James Harris PhD, who presented it at the Center for Business Intelligence conference titled *6^th ^Forum on Predictive ADME/Tox *held in Washington, DC September 27–29, 2006 and is adapted from data that appeared in Arun K Mandagere and Barry Jones. Prediction of Bioavailability. In (Eds) Han van de Waterbeemd, Hans Lennernäs, Per Artursson, and Raimund Mannhold. *Drug Bioavailability: Estimation of Solubility, Permeability, Absorption and Bioavailability (Methods and Principles in Medicinal Chemistry) *Wiley-VCS 2003. P444–60.

Figures 1 and 2 illustrate graphically our contention that animals are not predictive. Both figures chart bioavailability data from three species of animals and compare it to data from humans. (Bioavailability is usually defined as the fraction of a drug that reaches the systemic circulation and reflects a number of different variables. Regardless of the correlation or lack thereof of the variables, the bioavailability of the drug is the final determinant of how much drug presents to the receptor or active site.) Figure 1 was compiled by Harris from a paper by Grass and Sinko in *Advanced Drug Delivery Reviews*. As the reader can see the bioavailability of various drugs is measured in humans and three species of animals (representing primates, rodents and dogs) and the results plotted. Some of the drugs that showed high levels of bioavailability in dogs had very low levels in humans and vice-versa. This was true regardless of drug or species. Some levels did correlate between species but as a whole there was no correlation between what a drug did in humans and what it did in any given animal species or any combination thereof.

Figure 2 was complied by Harris from a book section by Mandagere and Jones in the book *Drug Bioavailability: Estimation of Solubility, Permeability, Absorption and Bioavailability (Methods and Principles in Medicinal Chemistry) *and made the same measurements and reached the same conclusions as did Grass and Sinko.

As you can see there is little correlation between animal and human data. In some cases human bioavailability is high when bioavailability in dogs is high but in other cases dogs and humans vary considerably. The patterns exhibited by both are what are frequently referred to as a shotgun pattern; meaning that if one fired a shotgun full of bird shot at a target one would see the same pattern. No precision and no accuracy. The pattern is also referred to as a scattergram, meaning that the pattern is what one would expect from random associations.

The above illustrates why eliminating drugs in development based on animal tests presents problems. Sankar in *The Scientist *2005:

The typical compound entering a Phase I clinical trial has been through roughly a decade of rigorous pre-clinical testing, but still only has an 8% chance of reaching the market. Some of this high attrition rate is due to toxicity that shows up only in late-stage clinical trials, or worse, after a drug is approved. Part of the problem is that the toxicity is assessed in the later stages of drug development, after large numbers of compounds have been screened for activity and solubility, and the best produced in sufficient quantities for animal studies.

Howard Jacob notes that rats and humans are 90% identical at the genetic level. However, the majority of the drugs shown to be safe in animals end up failing in clinical trials. "There is only 10% predictive power, since 90% of drugs fail in the human trials" in the traditional toxicology tests involving rats. *Conversely, some lead compounds may be eliminated due to their toxicity in rats or dogs, but might actually have an acceptable risk profile in humans *[39]. (Emphasis added.)

Again, for some this alone would settle the prediction question. But we continue.

Sensitivity is not the same prediction. While it is true that that all known human carcinogens that have been adequately studied have been shown to be carcinogenic in at least one animal species [40-42], it is also true that an irreverent aphorism in biology known as Morton's Law states: "If rats are experimented upon, they will develop cancer." Morton's law is similar to Karnofsky's law in teratology, which states that any compound can be teratogenic if given to the right species at the right dosage at the right time in the pregnancy. The point being that it is very easy to find positive results for carcinogenicity and teratogenicity; a high sensitivity. Nonetheless, this is meaningless without also knowing specificity, positive predictive value, and negative predictive value.

### Carcinogenesis

How well do animal models predict carcinogenesis? Possible carcinogens are listed in the Integrated Risk Information System (IRIS) chemicals database managed by the Environmental Protection Agency (EPA). According to Knight et al. [43] as of 1 January 2004, IRIS was unable to classify the carcinogenic status of 93 out of 160 chemicals that had been evaluated only by animal tests. The World Health Organisation also classifies chemicals according to carcinogenicity via the International Agency for Research on Cancer (IARC).

Knight et al. wrote in 2006:

For the 128 chemicals with human or animal data also assessed by the human carcinogenicity classifications were compatible with EPA classifications only for those 17 having at least limited human data (p = 0.5896). For those 111 primarily reliant on animal data, the EPA was much more likely than the IARC to assign carcinogenicity classifications indicative of greater human risk (p < 0.0001) [43].

This discrepancy is troublesome. Knight et al. discussed a study in 1993 by Tomatis and Wilbourn [44]. Tomatis and Wilbourn surveyed the 780 chemical agents or exposure circumstances listed within Volumes 1–55 of the IARC monograph series [45]. They found that "502 (64.4%) were classified as having definite or limited evidence of animal carcinogenicity, and 104 (13.3%) as definite or probable human carcinogens ... around 398 animal carcinogens were considered not to be definite or probable human carcinogens."

Knight et al. continue:

... based on these IARC figures, the positive predictivity of the animal bioassay for definite probable human carcinogens was only around 7% (104/502), while the false positive rate was a disturbing 79.3% (398/502) [43].

More-recent IARC classifications indicate little movement in the positive predictivity of the animal bioassay for human carcinogens. By January 2004, a decade later, only 105 additional agents had been added to the 1993 figure, yielding a total of 885 agents or exposure circumstances listed in the IARC Monographs [46]. Not surprisingly the proportion of definite or probable human carcinogens resembled the 1993 figure of 13.3%. By 2004, only 9.9% of these 885 were classified as definite human carcinogens, and only 7.2% as probable human carcinogens, yielding total of 17.1%.

Haseman [47] published a study in 2000 in which he revealed that 250 (53.1%) of chemicals in the NTP carcinogenicity database were carcinogenic in at least one sex-species group. He concluded that the actual number posing a significant carcinogenic risk to humans was probably far lower. Approximately half of all chemicals tested on animals and included in the comprehensive Berkeley-based potency carcinogenic database (CPDB) were carcinogenic [48].

Knight et al. conclude:

If a risk-avoidance interpretation is used, in which any positive result in male or female mice or rats is considered positive, then nine of the 10 known human carcinogens among the hundreds of chemicals tested by the NTP are positive, but so are an implausible 22% of all chemicals tested. If a less risk-sensitive interpretation is used, whereby only chemicals positive in both mice and rats are considered positive, then only three of the six known human carcinogens tested in both species are positive. The former interpretation could result in the needless denial of potentially useful chemicals to society, while the latter could result in widespread human exposure to undetected human carcinogens [43].

At this point in the debate, some will state that animal models can be useful in science and scientific research and attempt to conflate the word *predict *with the word *useful*. This is disingenuous for many reasons. First, *useful *is too ambiguous to mean anything. Useful to whom? Useful how? Almost anything can be useful in some sense of the word. If someone gets paid to engage in fortune telling then fortune telling is very useful to that person. Whether it can be used to predict the future is an entirely different question. We do not deny animal models can be quite *useful *in certain circumstances but this has nothing to do with whether they are predictive. Second, this is an example of bait and switch; sell animal models as predictive for humans then justify their use, since they are not predictive, because they are *useful*. Freeman and St Johnston illustrate this type of disingenuousness when they state:

Many scientists who work on model organisms, including both of us, have been known to contrive a connection to human disease to boost a grant or paper. It's fair: after all, the parallels are genuine, but the connection is often rather indirect. More examples will be discussed in later chapters [49].

Third, predict has a very specific meaning in science, indeed the concept of prediction is one thing that separate science from pseudoscience. By conflating *useful *and *predict *we diminish the respectability of science in general putting it more on the level of selling used cars. Finally, we again acknowledge that studying animals can lead to new knowledge. This point is not in dispute.

### Thalidomide

Let us take and in depth look at one drug and the animal tests that *could *have been performed and evaluate what we would have learned from them. There are many examples of animal models giving results at extreme variance from humans and even from each other; thalidomide being but one, but thalidomide occupies a special place in history so we will use it. Thalidomide was a sedative prescribed to pregnant women in the late 1950 and early 1960s. The children of some of these women were born without limbs, a condition known as phocomelia. Could the thalidomide tragedy have been predicted and prevented on the basis of animal experimentation as Gad [29] and others have claimed? Consider the evidence. Schardein who has studied this tragedy has observed:

In approximately10 strains of rats, 15 strains of mice, 11 breeds of rabbits, 2 breeds of dogs, 3 strains of hamsters, 8 species of primates and in other such varied species as cats, armadillos, guinea pigs, swine and ferrets in which thalidomide has been tested, teratogenic effects have been induced only occasionally [50].

We remind the reader that these results, and those below were from tests performed after thalidomide's affects had been observed in humans. Schardein also observes:

It is the actual results of teratogenicity testing in primates which have been most disappointing in consideration of these animals' possible use as a predictive model. While some nine subhuman primates (all but the bushbaby) have demonstrated the characteristic limb defects observed in humans when administered thalidomide, the results with 83 other agents with which primates have been tested are less than perfect. Of the 15 listed putative human teratogens tested in nonhuman primates, only eight were also teratogenic in one or more of the various species [51].

Manson and Wise summarized the thalidomide testing as follows:

An unexpected finding was that the mouse and rat were resistant, the rabbit and hamster variably responsive, and certain strains of primates were sensitive to thalidomide developmental toxicity. Different strains of the same species of animals were also found to have highly variable sensitivity to thalidomide. Factors such as differences in absorption, distribution, biotransformation, and placental transfer have been ruled out as causes of the variability in species and strain sensitivity [52].

Could the use of animal models have predicted thalidomide's adverse affects? Even if all the animals mentioned above were studied the answer is no. Different species showed a wide variety of responses to thalidomide. Once again, if you bet on enough horses you will probably find a winner or if you cherry pick the data you will find a winner. In the present case of thalidomide, human effects were already known so cherry picking is easy. The animal models for thalidomide discussed above were aimed at retroactively simulating *known *human effects. Even then not many animal models succeeded. If the human effects were *unknown*, what would the case have looked like from the standpoint of prediction? In this case, to pursue the horse racing analogy, we would have numerous horses to bet on without any idea which one would win. Certainly one will win (which is not a given when testing on animals in hopes of reproducing or guessing human response), but which one? We cannot know that until after the fact so how do we judge *prospectively *which horse to wager on or which animal model to choose? Which model species were relevant to the human case in advance of the gathering of human data? This is by no means a trivial question as evolutionary closeness does not increase the predictive value of the model. Caldwell points out that relatively small biological differences between test subjects can lead to very different outcomes:

It has been obvious for some time that there is generally no evolutionary basis behind the particular drug metabolizing ability of a particular species. Indeed, among rodents and primates, zoologically closely related species exhibit markedly different patterns of metabolism [53].

The thalidomide case illustrates why the overarching hypothesis that animals are predictive for humans is wrong. Again, this overarching hypothesis is in contrast to using animals as heuristic devices where the hypotheses drawn from them must be tested.

Even if we retrospectively picked all the animals that reacted to thalidomide as humans did, we still could not say these animals predicted human response as their history of agreeing with human response to other drugs varied considerably. Prediction vis-à-vis drug testing and disease research implies a track record. Single correct guesses are not predictions. Nonhuman primates are a good example of this. They more or less reacted to thalidomide as humans did (so we will give them the benefit of the doubt as corresponding to humans in this case). However, when tested with other drugs they *predicted *human response about as well as a coin toss. Add to all this the fact that all the animals whose offspring exhibited phocomelia consequent to the administration of thalidomide did so only after being given doses 25–150 times the human dose [54-56] and it does not appear that any animal, group of animals, or the animal model *per se *could have been used to predict thalidomide's teratogenicity in humans. (Ironically, it was the thalidomide tragedy that ushered in many of the regulatory requirements for using animals.)

Thalidomide's controversial history should not interfere with our analysis, as the history in question does not overlap with our issue. The controversy revolves around what animals were tested, whether pregnant animals were tested, what the drug company knew and when they knew it and so forth. This is immaterial, as we are analyzing the data as if it were available before releasing the drug. We are giving the animal model the maximum benefit of the doubt and what we find is that even if all the data available today had been available then, the decision to release the drug or not would not have been *informed *by animal tests. Karnofsky's law is relevant here. Any drug is teratogenic if given to the right animal at the right time. Given thalidomide's profile today, physicians would advise pregnant women not to take the drug, which is what physicians advise every pregnant woman about almost every nonlife-saving drug anyway, regardless of the results of animal tests.

The claim that thalidomide's affects were or could have been predicted by animals is an example of cherry picking the data.

### The quantitative/qualitative controversy

We now move on to the quantitative/qualitative controversy. There is a tendency on the part of some researchers to see all differences between species as mere quantitative differences – presumably differences that can be compensated for in the context of prediction. Vineis and Melnick:

However, we disagree with Shanks and Pyles about the usefulness of animal experiments in predicting human hazards. Based on the darwinian observation of inter-species and inter-individual variation in all biological functions, Shanks and Pyles suggest that animal experiments cannot be used to identify hazards to human health. We claim that while the activity of enzymes may vary among individuals and among species, this does not indicate that critical events in disease processes occurring after exposure to hazardous agents differ qualitatively between animal models and humans.... For the most part, differences in how laboratory animals and humans metabolize environmental agents, or in the interactions of these agents with molecular targets (e.g., DNA, enzymes, or nuclear receptors), are quantitative in nature [27].

This is very much a Newtonian way of thinking and it ignores the effects of biological evolution and the fact that animals are complex systems.

Toxicologists have known for a long time that species differences may be *quantitative *or *qualitative *[53,57]. Consider a model substrate such as phenol. Humans and rats excrete phenol through two pathways, sulfate conjugation and glucuronic acid conjugation. There is a quantitative difference between humans and rats since the ratios of sulfate conjugation to glucuronic acid conjugation are different in each species. But there are qualitative differences too. Cats are incapable of glucuronic acid conjugation, and must excrete phenol via the sulfate pathway. For pigs the reverse is true, they cannot use the sulfate pathway, and must rely on glucuronic acid conjugation. (It is worth noting that there are at least seven metabolic pathways that are unique to primates – for example the aromatization of quinic acid [57].)

One lesson to be drawn from this example is that even if the same function is achieved by two species (e.g., excretion of phenol), it does not follow that they are doing so by the exact same underlying causal mechanisms. In the context of toxicology or pharmacology, these mechanistic differences can be important in assessing safety as well as pharmacological utility.

### Other voices

We are not the only ones concerned about the predictive power of animal models. The scientific community itself is not marching in lock step when it comes to the predictive utility of animal models. We will take a moment to examine what some of these scientists actually say about the power of animal models to predict human responses. The following quotes from scientists (and the above quotes from Leavitt and Sankar), of course, prove nothing in the sense of mathematical proof, they nevertheless provide a window into the thinking of people well versed in the field and as such a reasonable person should take them seriously. They should give pause to those who think that the prediction issue is one where there is no reasonable controversy.

R.J. Wall and M. Shani observe:

The vast majority of animals used as models are used in biomedical preclinical trials. The predictive value of those animal studies is carefully monitored, thus providing an ideal dataset for evaluating the efficacy of animal models. On average, the extrapolated results from studies using tens of millions of animals fail to accurately predict human responses ... We conclude that it is probably safer to use animal models to develop speculations, rather than using them to extrapolate [58].

Curry points out:

The failure, in the clinic, of at least fourteen potential neuroprotective agents expected to aid in recovery from stroke, after studies in animal models had predicted that they would be successful, is examined in relation to principles of extrapolation of data from animals to humans [59].

The above proves two things. 1. At least some members of the animal experimentation community do know what the word predict means. 2. They also know animal models are not predictive. Their analysis and conclusions, which revealed the failure of animal models, was neither new nor surprising. History reveals the same.

Discrepancies between animal-human studies and even animal-animal studies date back centuries. Percival Pott showed coal tar was carcinogenic to humans in 1776. Yamagiwa and Ichikawa showed it was carcinogenic in some animals in 1915. But even then, rabbits did not respond as mice [60]. In 1980 there were roughly sixteen-hundred known chemicals that caused cancer in mice and rodents, but only approximately fifteen known to cause cancer in humans [61]. The Council on Scientific Affairs publishing in the *Journal of the American Medical Association *in 1981 stated:

*The Council's consultants agree that to identify carcinogenicity in animal tests does not per se predict either risk or outcome in human experience *... the Council is concerned about the hundreds of millions of dollars that are spent each year (both in the public and private sectors) for the carcinogenicity testing of chemical substances. The concern is particularly grave in view of the questionable scientific value of the tests when used to predict human experience [62]. (Emphasis added.)

David Salsburg of Pfizer wrote in 1983 that a report by the National Cancer Institute that examined 170 chemicals concluded that lifetime feeding studies using rodents lacked sensitivity and specificity. He stated:

If we restrict attention to long term feeding studies with mice or rats, only seven of the 19 human non-inhalation carcinogens (36.8%) have been shown to cause cancer. If we consider long term feeding or inhalation studies and examine all 26, only 12 (46.2%) have been shown to cause cancer in rats or mice after chronic exposure by feeding or inhalation. Thus the lifetime feeding study in mice and rats appears to have less than a 50% probability of finding known human carcinogens.*On the basis of probability theory, we would have been better off to toss a coin *[63]. (Emphasis added.)

Should we discard every drug that causes cancer in animals? Acetaminophen, chloramphenicol, and metronidazole are known carcinogens in some animal species [64,65]. Phenobarbital and isoniazid are carcinogens in rodents[60,66,67]. Does this mean they never should have been released to the market? Diphenylhydantoin (phenytoin) is carcinogenic to humans but not rats and mice [68-70]. Occupational exposure to 2-naphthylamine appears to cause bladder cancer in humans. Dogs and monkeys also suffer bladder cancer if exposed to 2-naphthylamine orally and mice suffer from hepatomas. It does not appear to have carcinogenic properties in rats and rabbits. These are qualitative differences due to differences in metabolism of aromatic amines [71]. It also appears that fewer genetic, epigenetic, or gene expression events are needed to induce cancer in rodents than are needed to induce cancer in humans [72-74]. (A good review of species differences in relation to carcinogenesis and why they exist is Anisimov et al. [72].)

Intraspecies differences also exist. Clofibrate, nafenopin, phenobarbital, and reserpine cause cancer in old but not young rats [68,75].

Should the above drugs that caused cancer in some animal have been banned? If the null hypothesis is that there is no association between animal carcinogens and human carcinogens strong enough so the animal model can be said to be predictive, then we see much evidence to support the null hypothesis but very little if any to refute it.

The point to be made here is that there are scientists (rather more than we have space to mention) who question the predictive and/or clinical value of animal-based research and history is on their side. As noted above, the opinions of scientists prove nothing in and of itself. Further, some of what we have presented could be dismissed as anecdotes but this would be a mistake. First, the studies referenced in the previous section are just that, scientific studies not anecdotes. Second, the examples presented are referenced, anecdotes are not (unless they are case reports and we must remember that thalidomide's downfall started as a series of case reports). But again we must ask where the burden of proof lies? We believe the second law of thermodynamics because there has never been an example of where it was wrong. Just one such example would falsify the law. If the animal model community claims the animal model is predictive, then they must explain the examples and studies that reveal it was not. Examples such as case reports count when disproving an assertion, especially when they are then supported with studies, but cannot be used by those making the claim as proof for their overarching hypothesis. That is how science works. We did not make the rules. In summary there is ample evidence to question, if not disprove entirely, the overarching hypothesis that animal models are predictive for humans.

To take the argument one step further, we must ask what conditions ought to be satisfied if animals are to serve as predictors of human biomedical phenomena. This is a question concerning theory and come under the heading of philosophy of science.

### Theory

The requirements that need to be satisfied to get genuine causal predictions (as opposed to mere correlations) about members of one species on the basis of test results on members of another species are very difficult to satisfy (and may at best only be approximated in a few cases).

Models or a modality claiming predictability assumes identical causal properties. As researchers Carroll and Overmier explain in their recent book *Animal Research and Human Health *[76], and as LaFollette and Shanks also do in *Brute Science*[3], animals in biomedical research are frequently used as causal analogical models (CAMs). If the heart pumps blood in a chimpanzee, then we reason by analogy it will pump blood in humans also. If fen-phen is safe for the hearts of animals we reason by analogy it will be safe for human hearts as well [77]. Carroll and Overmier state:

When the experimenter devises challenges to the animal and studies a causal chain that, through analogy, can be seen to parallel the challenges to humans, the experimenter is using an animal model [76].

These are examples of using animals as CAMs or predictive models according to the traditionally used definition of the word prediction and as used by us in this article. We will discuss CAMs more fully in the section on theory.

Animal models in this sense involve causal analogical reasoning. First, what is a causal analogical model (CAM) and how does it involve causal analogical reasoning? The first condition that must be met in order for a thing to be considered a CAM is this: "X (the model) is similar to Y (the object being modelled) in respects {*a...e*}." If "X has additional property *f*, then while *f *has not been observed directly in Y, likely Y also has property *f *[3]." This latter claim is something that needs to be tested. In the present case it means the gathering of human data.

This first condition is not enough. For instance chimpanzees and humans have (a) an immune system, (b) have 99% of their DNA in common with humans, (c) contract viruses, etc. HIV reproduces very slowly in chimpanzees. We therefore expect HIV to reproduce slowly in humans. [3]. So if HIV replicates slowly in chimpanzees, animal experimenters reason *by analogy *that it will do the same in humans. This turns out to be false.

CAMs must satisfy two further conditions: (1) the common properties (*a, ..., e*) must be causal properties which (2) are causally connected with the property (*f*) we wish to project – specifically, (*f*) should stand as the cause(s) or effect(s) of the features (*a, ..., e*) in the model. When animals are used as causal analogical models the reasoning process taking us from results in the model to the system modelled is called *causal analogical reasoning *[3].

But it is not enough simply to point to similarities to justify cross-species extrapolation in the context of causal analogical reasoning. In complex, interactive systems such as organisms, we need to know whether there are relevant causal differences, i.e., causal disanalogies (with respect to mechanisms and pathways) that compromise the usefulness of the analogical reasoning. In other words, for a CAM to be predictive, there should be no causally-relevant disanalogies between the model and the thing being modeled. For example, there must be no properties {*g, h, i*} unique to either the model or the object modelled that causally interact with the common properties {*a...e*}, since such properties will likely compromise the predictive utility of the model.

The idea here is an old one. It concerns *causal determinism *– a concept that has played a fundamental role in the development of modern science. Causal determinism rests on two basic principles: (1) *The Principle of Causality*, according to which all events have causes; and (2) *The Principle of Uniformity*, according to which, for qualitatively identical system, all other things being equal, same cause is always followed by same effect.

These ideas played a role in our earlier discussion of Newtonian mechanics at the beginning of this essay. In a way, the whole issue of prediction comes down to the principle of uniformity. Are the animals used to make predictions about humans qualitatively identical to humans once allowances have been made for difference in body weight or surface area? No reasonable person who understands evolutionary biology, and who knows, for example, that rodents and humans have taken very different evolutionary trajectories since the lineages leading to modern humans and rodents, respectively, diverged over seventy million years ago, will expect qualitative identity. But perhaps qualitative identity is an ideal that can only be approximated. Are humans and their animal models sufficiently similar for approximate predictions to be made? The numerous studies referenced above, say no. Why is this the case?

Vertebrates are evolved complex systems. Such systems may manifest different responses to the same stimuli due to: (1) differences with respect to genes/alleles present; (2) differences with respect to mutations in the same gene (where one species has an ortholog of a gene found in another); (3) differences with respect to proteins and protein activity; (4) differences with respect to gene regulation; (5) differences in gene expression; (6) differences in protein-protein interactions; (7) differences in genetic networks; (8) differences with respect to organismal organization (humans and rats may be intact systems, but may be differently intact); (9) differences in environmental exposures; and last but not least; (10) differences with respect to evolutionary histories. These are some of the important reasons why members of one species often respond differently to drugs and toxins, and experience different diseases. These ten facts alone would be sufficient for some to conclude that animal models cannot be predictive for human; that transspecies extrapolation is impossible vis-à-vis drug response and disease research especially when analyzed in lights of the standards society today demands. (And the standards not set too high. If you think they are ask yourself if, had you taken rofecoxib and been harmed, would you have accepted a .99 PPV as acceptable?)

In biomedicine we do not have the mathematician's luxury of modeling humans and rodents by beginning, "let humans and rodents be spheres." If only it were that simple. Instead, what we do have are a lot of theoretical grounds for questioning the predictive utility of animal models. But of course, such theoretical reasoning may be dismissed as being just that. The real question is one of evidence. We have examined the evidence against and found it compelling but we should now examine the evidence cited as supporting the predictive nature of animals. We will now turn our attention to the famous Olson study, which many suppose to have settled the matters at hand firmly on the side of animal model being predictive for humans.

### The Famous Olson Study

The Olson study [78] purports (and has certainly been cited in this regard) to provide evidence of the vast predictive utility of animal models in assessing human toxicity. In response to an article by Shanks et al. [79] Conn and Parker quoted the Olson study stating:

The authors have simply overlooked the classic study (Olson, Harry, et al.., 2000. "Concordance of the Toxicity of Pharmaceuticals in Humans and in Animals." *Regul Toxicol Pharmacol*32, 56–67) that summarizes the results from 12 international pharmaceutical companies on the predictivity of animal tests in human toxicity. While the study is not perfect, the overall conclusion from 150 compounds and 221 human toxicity events was that animal testing *has *significant predictive power to detect most – but not all – areas of human toxicity [80]. (Emphasis theirs.)

We encourage the reader to examine the Olson Study in its entirety. Here we include some important representative paragraphs from the Olson study and our commentary will follow. We apologize for the length of the quote but due to the importance many place on the paper, we believe a thorough examination is justified.

This report summarizes the results of a multinational pharmaceutical company survey and the outcome of an International Life Sciences Institute (ILSI) Workshop (April 1999), which served to better understand *concordance of the toxicity *of pharmaceuticals observed in humans with that observed in experimental animals. The Workshop included representatives from academia, the multinational pharmaceutical industry, and international regulatory scientists.*The main aim of this project was to examine the strengths and weaknesses of animal studies to predict human toxicity (HT). The database was developed from a survey which covered only those compounds where HTs were identified during clinical development of new pharmaceuticals*, determining whether animal toxicity studies identified concordant target organ toxicities in humans ...

The results showed the *true positive HT concordance rate of 71% *for rodent and nonrodent species, with nonrodents alone being predictive for 63% of HTs and rodents alone for 43%. The highest incidence of overall concordance was seen in hematological, gastrointestinal, and cardiovascular HTs, and the least was seen in cutaneous HT. *Where animal models, in one or more species, identified concordant HT, 94% were first observed in studies of 1 month or less in duration*. These survey results support the value of in vivo toxicology studies to predict for many significant HTs associated with pharmaceuticals and have helped to identify HT categories that may benefit from improved methods ...

The primary objective was to examine how well toxicities seen in preclinical animal studies would predict actual human toxicities for a number of specific target organs using a database of existing information ...

**Figure 3:**
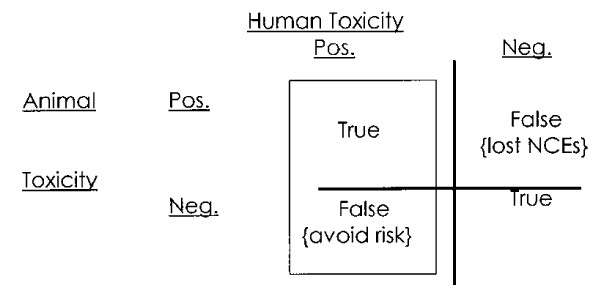
**Olsen figure 1**.

*Although a considerable effort was made to collect data that would enable a direct comparison of animal and human toxicity, it was recognized from the outset that the data could not answer completely the question of how well animal studies predict overall the responses of humans. To achieve this would require information on all four boxes in Fig. *1*, and this was not practicable at this stage*. The magnitude of the data collection effort that this would require was considered impractical at this stage. *The present analysis is a first step, in which data have been collected pertaining only to the left column of Fig. *1*: true positives and false negatives*. [See figure 3.] By definition, therefore the database only contains compounds studied in humans (and not on those that never reached humans because they were considered too toxic in animals or were withdrawn for reasons unrelated to toxicity). Despite this limitation, it was deemed useful to proceed in the expectation that any conclusions that emerged would address some of the key questions and focus attention on some of the strengths and weaknesses of animal studies ...

A working party of clinicians from participating companies developed criteria for "significant" HTs to be included in the analysis. For inclusion a HT (a) had to be responsible for termination of development, (b) had to have resulted in a limitation of the dosage, (c) had to have required drug level monitoring and perhaps dose adjustment, or (d) had to have restricted the target patient population. The HT threshold of severity could be modulated by the compound's therapeutic class (e.g., anticancer vs anti-inflammatory drugs). In this way, *the myriad of lesser "side effects" that always accompany new drug development but are not sufficient to restrict development were excluded*. The judgments of the contributing industrial clinicians were final as to the validity of including a compound. The clinical trial phase when the HT was first detected and whether HT was considered to be pharmacology-related was recorded. HTs were categorized by organ system and detailed symptoms according to standard nomenclature (COSTART, National Technical Information Service, 1999) ...

**Figure 4:**
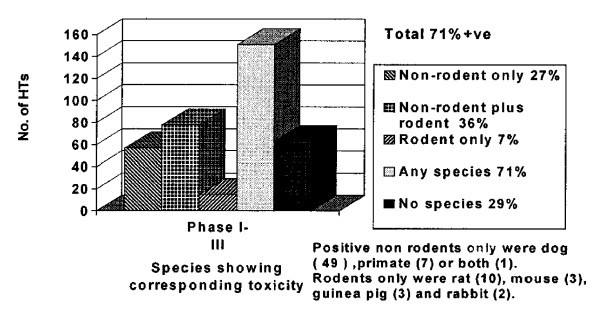
**Olsen figure 3**.

Concordance by one or more species: Overall and by HT. *Overall, the true positive concordance rate (sensitivity) was 70% for one or more preclinical animal model species (either in safety pharmacology or in safety toxicology) showing target organ toxicity in the same organ system as the HT *[Fig. 4].

*This study did not attempt to assess the predictability of preclinical experimental data to humans*. What it evaluated was the concordance between adverse findings in clinical data with data which had been generated in experimental animals (preclinical toxicology) [78]. (Emphasis added.)

**Figure 5:**
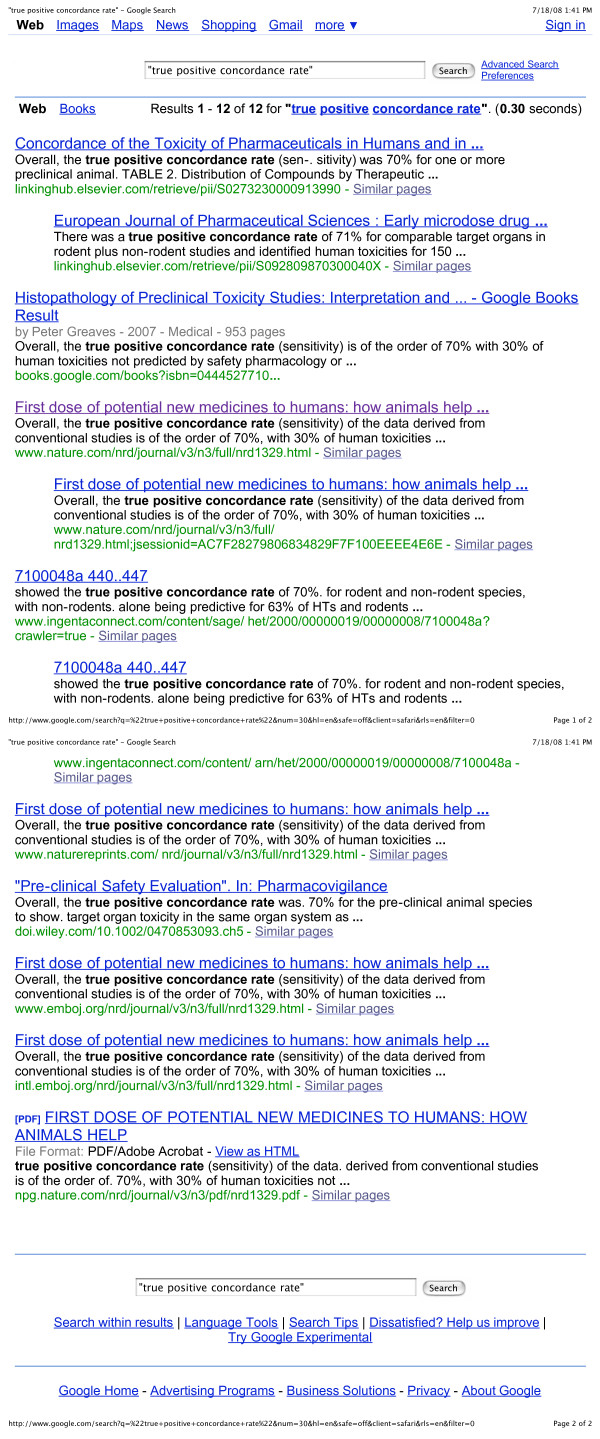
**Google results**.

The Olson Study, as noted above, has been employed by researchers to justify claims about the predictive utility of animal models. However we think there is much less here than meets the eye. Here's why:

1. The study was primarily conducted and published by the pharmaceutical industry. This does not, in and of itself, invalidate the study. However, one should never lose sight of the fact that the study was put together by parties with a vested interest in the outcome. If this was the only concern, perhaps it could be ignored, however, as we will now show, there are some rather more serious flaws.

2. The study says at the outset that it is aimed at measuring the predictive reliability of animal models. Later the authors concede that their methods are not, as a matter of fact, up to this task. This makes us wonder how many of those who cite the study have actually read it in its entirety.

3. The authors of the study invented new statistical terminology to describe the results. The crucial term here is "true positive concordance rate" which sounds similar to "true predictive value" (which is what should have been measured, but was not). A Google search on "true positive concordance rate" yielded twelve results (counting repeats), all of which referred to the Olson Study (see figure 5). At least seven of the twelve Google hits qualified the term "true positive concordance rate" with the term "sensitivity" – a well-known statistical concept. In effect, these two terms are synonyms. Presumably the authors of the study must have known that "sensitivity" does not measure "true predictive value." In addition you would need information on "specificity" and so on, to nail down this latter quantity. If all the Olson Study measured was sensitivity, its conclusions are largely irrelevant to the great prediction debate.

4. Any animals giving the same response as a human was counted as a positive result. So if six species were tested and one of the six mimicked humans that was counted as a positive. The Olson Study was concerned primarily not with prediction, but with retroactive simulation of antecedently know human results.

5. Only drugs in clinical trials were studied. Many drugs tested do not actually get that far because they fail in animal studies.

6. "...the myriad of lesser "side effects" that always accompany new drug development but are not sufficient to restrict development were excluded." A *lesser *side effect is one that affects someone else. While hepatotoxicity is a major side effect, lesser side effects (which actually matter to patients) concern profound nausea, tinnitus, pleuritis, headaches and so forth. We are also left wondering whether there was any independent scientific validity for the criteria used to divide side effects into major side effects and lesser side effects.

7. Even if all the data is good – and it may well be – sensitivity (i.e. true positive concordance rate) of 70% does not settle the prediction question. Sensitivity is not synonymous with prediction and even if a 70% positive prediction value rate is assumed, when predicting human response 70% is inadequate. In carcinogenicity studies, the sensitivity using rodents may well be 100%, the specificity, however, is another story. That is the reason rodents cannot be said to predict human outcomes in that particular biomedical context.

The Olson Study is certainly interesting, but even in its own terms it does not support the notion that animal models are predictive for humans. We think it should be cited with caution. A citation search (also performed with Google on 7/23/08) led us to 114 citations for the Olson paper. We question whether caution is being used in all these citations.

## Conclusion

Mark Kac stated, "A proof is that which convinces a reasonable man." Even though the burden of proof is not on us to prove animal models are *not *predictive, we believe we have presented a proof that would convince a reasonable man.

There are both quantitative and qualitative differences between species. This is not surprising considering our current level of knowledge vis-à-vis evo devo, gene regulation and expression, epigenetics, complexity theory, and comparative genomics. Hypotheses generate predictions, which can be then proven true or false. Predict has a very distinct meaning in science and according to some is the foundation of science itself. Prediction does not mean retrospectively finding one animal that responded to stimuli like humans and therefore saying that the animal predicted human response nor does it mean cherry picking data nor does it mean occasionally getting the right answer.

When a concept such as "Animal models can predict human response" is accepted as true, it is not functioning as a hypothesis. We have referred to this as an overarching hypothesis but could have easily referred to it as an unfounded assumption. An assumption or overarching hypothesis might in fact be true but its truth must be proven. If a modality such as animal testing or using animals to predict pathophysiology in human disease is said to be a predictive modality, then any data generated from said modality should have a very high probability of being true in humans. Animal models of disease and drug response fail this criterion.

In medicine, even positive predictive values of .99 may be inadequate for some tests and animal models do not even roughly approximate that. Therefore, animal models are not predictors of human response. Some animals do occasionally respond to stimuli as do humans. However, how are we to know prospectively which animal will mimic humans? Advocates who maintain animals are predictive confuse sensitivity with prediction. Animals as a group are extremely sensitive for carcinogenicity or other biological phenomena. Test one hundred different strains or species and one is very likely to react like humans. But the specificity is very low; likewise the positive and negative predictive values. (Even if science did decide to abandon the historically correct use of the word predict, every time an animal-model advocate said animal species × predicted human response Y, she would also have to admit that animal species A, B, C, D, E and so forth predicted incorrectly. Thus justifying the use of animals because animal models *per se *to make our drug supply safer or predict facts about human disease would not be true.)

Some have suggested we should not criticize animal models unless we have better suggestions for research and testing [27]. It is not incumbent upon us to postpone criticizing animal models as not being predictive until predictive models such as *in silico*, *in vitro *or *in vivo *are available. Nor is it incumbent upon us to invent such modalities. Astrology is not predictive for foretelling the future therefore we criticize such use even though we have no notion of how to go about inventing such a future-telling device.

Some have also suggested that animal models may someday be predictive and that we should so acknowledge. While this is true in the sense that anything is possible it seems very unlikely, as genetically modified organisms have been seen to suffer the same prediction problems we have addressed [16,81-87] and, as mentioned different humans have very different responses to drugs and disease. Considering our current understanding of complex systems and evolution it would be surprising if one species could be used to predict outcomes in another at the fine-grained level where our study of disease and drug response is today and to the accuracy that society demands from medical science.

There are direct and indirect consequences to this misunderstanding of what prediction means. If we did not allow on the market any chemical or drug that causes cancer, or is teratogenic, or causes severe side effects in any species, then we would have no chemicals or drugs at all. Furthermore, there is a cost to keeping otherwise good chemicals off the market. We lose: treatments perhaps even cures; the income that could have been generated; and new knowledge that could have been gained from learning more about the chemical. These are not insignificant downsides. Since we now understand vis-à-vis personalized medicine that even humans differ in their response to drugs and disease and hence one human cannot predict what a drug will do to another human, it seems illogical to find models that are predictive using completely different species from humans. If we truly want predictive tests and research methods (and we do), it would seem logical to start looking intraspecies not interspecies.

## Competing interests

The authors declare that they have no competing interests.

## Authors' contributions

All authors contributed equally and have read and approved the manuscript.

## About the authors

Niall Shanks, PhD is the Curtis D. Gridley Professor in the History and Philosophy of Science at Wichita State University where he is also Professor of Philosophy, Adjunct Professor of Biological Sciences, Adjunct Professor of Physics, and Associate Member of the Graduate Faculty. He is the President (2008–9) of the Southwest and Rocky Mountain Division of the American Association for the Advancement of Science. He received a PhD in Philosophy from the University of Alberta.

Ray Greek, MD completed medical school at the University of Alabama in Birmingham and a residency in anesthesiology at the University of Wisconsin-Madison. He taught at the University of Wisconsin and Thomas Jefferson University in Philadelphia. He is now the president of the not for profit Americans For Medical Advancement http://www.curedisease.com.

Jean Greek, DVM completed veterinary school at the University of Wisconsin-Madison and a residency in dermatology at the University of Pensylvania. She taught at the University of Missouri and is now in private practice.

## Acknowledgements

This paper has been made possible in part by a grant from the National Anti-Vivisection Society of Chicago, IL http://www.navs.org to Americans For Medical Advancement http://www.curedisease.com. Americans For Medical Advancement is a not for profit educational organization whose position on the use of animals is summarized nicely in this article. While NAVS opposes all animal-based research, AFMA does not. All authors are officers or directors of Americans For Medical Advancement.
